# Standardization of Solvent Type and Extraction Time for Lipid Extraction From Brewers' Spent Grain (BSG) by Soxhlet Method

**DOI:** 10.1002/fsn3.71398

**Published:** 2026-01-12

**Authors:** Veeramani Karuppuchamy, Osvaldo Campanella

**Affiliations:** ^1^ Department of Food Science and Technology The Ohio State University Columbus Ohio USA

**Keywords:** extraction time, organic solvents, Soxhlet method, spectroscopy, wet chemistry methods

## Abstract

Brewers' spent grain (BSG)'s potential use as a food ingredient is being evaluated, and thus, an accurate characterization of its proximate composition, particularly lipids, becomes essential. While the Soxhlet method is a widely recognized official standard test used for lipids measurement in food products, inconsistencies in solvent selection have led to variable and often no comparable results across studies. This is the first study that attempts to address that gap by systematically evaluating the impact of solvent type and extraction time on lipid yield from BSG. Five solvents and solvent mixtures, including acetone, ethanol, hexane, petroleum ether, and n‐hexane, were used for lipid extraction. In the initial phase, 5 g BSG was extracted using 125 mL of various solvents over 5 h. Lipid yields from BSG varied from 4.54% to 8.44% depending on the solvent type, in which ethanol yielded the highest lipid content. Based on these results, ethanol was selected for further studies on the effect of extraction time. Soxhlet extractions were performed using ethanol and it's binary mixtures with hexane and n‐hexane for 3, 5, and 7 h. The lipid yield from BSG varied from 7.66% to 8.44%, while the statistical analysis indicated no significant difference in lipid yield across the time range under studied conditions. Based on the findings from the study, it is recommended to use ethanol as a single solvent for 3‐h extraction, as the method balances efficiency and resource use. The results from this study is beneficial for the laboratories to choose the proper solvent and extraction time for measuring lipids in BSG using the Soxhlet method.

## Graphical abstract

Impact of solvent type and extraction time on lipid yield from brewers spent grain.

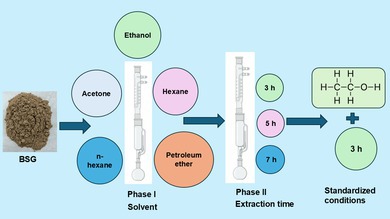

## 1 Introduction

Brewers' spent grains (BSG) is a major byproduct of breweries accounting for 85% of total waste. In its dried form, BSG is an excellent source of nutrients like dietary fiber and proteins. The exact amount of nutrients in BSG vary depending on the variety of the beer type, barley used in production, and fermentation conditions, etc. (Mussatto et al. 2006). From a circular economical perspective, many research studies have been conducted to explore the potential of BSG in food formulations. One of the major requirements for the incorporation of BSG is to quantify the major nutrients like proteins, lipids, and carbohydrates. Although instrument‐based techniques (secondary methods) are commonly used and preferred in the food industry to measure the composition of food components such as lipids, proteins, and moisture, calibration of these instruments must be developed and based on wet chemistry or reference methods (primary methods). Thus, the importance of wet chemistry methods in ensuring accurate and reliable measurements cannot be understated.

Reference methods for moisture and ash content involve the use of temperature and time. However, the measurement of lipids is very challenging compared to moisture, ash, or proteins. Soxhlet method is one of the well‐known reference methods for the estimation of lipids in food samples, and it has been approved by many regulatory agencies internationally. However, the efficiency of lipid extraction using the method is highly dependent on the choice of solvent and extraction time. This variability presents a challenge in achieving consistent and standardized results, highlighting the optimization and method refinement. In the case of BSG, many research studies have employed different solvents and extraction times as summarized in Table 1.

**TABLE 1:** Solvents used in the Soxhlet method for lipid extraction from BSG.

Sl. no	Solvent	Sample size (g)	Extraction time (h)	References
1	Diethyl ether	1	NR	Pires et al. (2012)
2	n‐hexane	30	6	Mallen and Najdanovic‐Visak (2018)
3	Chloroform	4	20	Connolly et al. (2013)
4	Heptane	NR	5	Niemi et al. (2012)
5	Acetone	0.50	8	del Río et al. (2013)
6	Hexane	5	6	Ferrentino et al. (2019)
7	n‐hexane	NR	12	Vieira et al. (2017)
8	Hexane	NR	NR	Guarda et al. (2021)
9	Hexane	NR	4	Kavalopoulos et al. (2021)
10	n‐hexane	2	5	Vieira et al. (2014)
11	Diethyl ether	3	14	Santos et al. (2003)
12	Ethanol	3	4	Moreira et al. (2012)
13	95% ethanol	3	6	Herbst et al. (2021)
14	Ethyl acetate	3	6	Herbst et al. (2021)
15	Acetone	3	6	Herbst et al. (2021)
16	n‐hexane	3	6	Herbst et al. (2021)
17	Petroleum ether	NR	NR	Parekh et al. (2017)
18	Isopropanol	NR	NR	Becker et al. (2021)
19	Ethanol	5	4	Ortiz et al. (2015)
20	97% ethanol	NR	6	de Crane d'Heysselaer et al. (2022)
21	96% ethanol	30	3	Bohnsack et al. (2011)
22	n‐hexane	30	3	Bohnsack et al. (2011)
23	Hexane	NR	NR	Alonso‐Riaño et al. (2022)
24	n‐hexane	10	3	Madubuike and Okolo (2016)
25	Petroleum ether	NR	NR	Ajanaku et al. (2011)
26	Petroleum ether	NR	NR	Bonifácio‐Lopes et al. (2020)

Abbreviation: NR, not reported.

Due to the complex nature of lipids present in samples, a careful selection of solvent must be performed. In addition to the solvent, extraction time is another factor that must be standardized. Extraction times varied from 3 to 20 h. While a shorter extraction time may not extract all lipids from the sample matrix, a very long extraction time might not be an effective approach, considering the time and energy consumption as well as the laboratory safety perspective due to the hazardous nature of many organic solvents.

To the best of the author's knowledge, no studies have been conducted to standardize the choice of solvent and extraction times for lipid extraction from BSG using the Soxhlet method. In this study, five solvents were selected based on their frequent use in previous Soxhlet‐based extractions of BSG. Reported extraction times in the literature range from 3 to 6 h. Therefore, the objective of this study is to evaluate frequently used solvents and extraction times to establish standardized parameters specific to BSG in the Soxhlet method.

## 2 Materials and Methods

### 2.1 Materials

#### 2.1.1 Brewers' Spent Grain (BSG)

Dried and milled brewers' spent grain (BSG) was provided by Regrained (Berkeley, CA, USA). BSG was stored in an airtight plastic container at ambient temperature until used for Soxhlet analysis. Moisture content was determined by drying the sample at 135°C for 2 h in a hot air oven (Binder Inc., Bohemia, NY, USA). The moisture content was 2.67% ± 0.08%. The particle size of BSG was 150 μm. In general, low‐moisture food products with are considered safe from a microbiological perspective, although there were a few instances of foodborne outbreaks associated with them (Karuppuchamy et al. 2024).

#### 2.1.2 Organic Solvents and Cellulose Thimbles

Acetone, hexane, petroleum ether, ethanol, and n‐hexane were purchased from Fisher Scientific (Fair Lawn, NJ, USA). The chemicals were stored in a flammable cabinet until use. Cellulose thimbles (33 mm × 80 mm) were procured from Fisher Scientific (Fair Lawn, NJ, USA). All chemicals used in the study were analytical grade. Selected properties of organic solvents used for extraction are given in Table 2.

**TABLE 2:** Properties of solvents selected in the current study.

Sl. no	Solvent	Polarity index	Boiling point (°C)	Density at 20°C (g/mL)
1	Acetone	5.1	56	0.790
2	Ethanol	5.2	78	0.789
3	Hexane	0.1	67–69.5	0.672
4	Petroleum ether	0.1	40–60; 60–80	0.640–0.680

*Source:* https://www.fishersci.co.uk/gb/en/scientific‐products/technical‐tools/summary‐key‐physical‐data‐solvents.html; https://research.cbc.osu.edu/turro.1/wp‐content/uploads/2017/02/solvent.miscibility.pdf.

### 2.2 Methods

This study was conducted in two phases. In phase I, lipid extraction was carried out for 5 h using different solvents and solvent mixtures. The solvent(s) or solvent mixtures yielding the highest lipid recovery were then selected for phase II, which evaluated the effects of extraction time.

#### 2.2.1 Effect of Single Solvent and Binary Mixtures

5 g of BSG was loaded in a cellulose thimble and placed within the extraction chamber of Soxhlet apparatus. A total of 125 mL of an organic solvent or a binary mixture was added to a 250 mL flask which was heated to its boiling point. Evaporated solvent or binary mixture was then cooled back to liquid state in the condensation chamber. Lipid extraction was carried out for 5 h during phase I. After 5 h extraction, the organic solvent or solvent mixture in the flask was removed using a rotary vacuum evaporator. The flasks with lipids were dried overnight at 50°C. Percent lipid content in BSG was calculated using Equation (1).

(1) $$\text{Lipids}\;\left(\%\right)=\frac{\left(\text{Weight of flask}+\text{lipids}\right)-\text{Weight of empty flask}}{\text{Weight of}\;\mathrm{BSG}\;\text{sample}\;}\times 100$$

#### 2.2.2 Effect of Extraction Time

Based on results from phase I, the solvent or binary mixture that yielded the highest lipid recovery was selected for a follow‐up study to assess the effect of extraction time. Thus, in phase II, Soxhlet extraction was conducted for 3 and 7 h.

### 2.3 Statistical Analysis

The statistical analysis was conducted using JMP Pro version 17 (SAS, Cary, NC, USA). Analysis of variance (ANOVA) was used to assess differences among treatments, and the Tukey's Honestly Significant Difference (HSD) test was applied for mean comparison. A *p* value less than 0.05 was considered statistically significant. All experiments were performed in triplicates, unless otherwise specified.

## 3 Results and Discussion

### 3.1 Effect of Single Solvent

**FIGURE 1:**
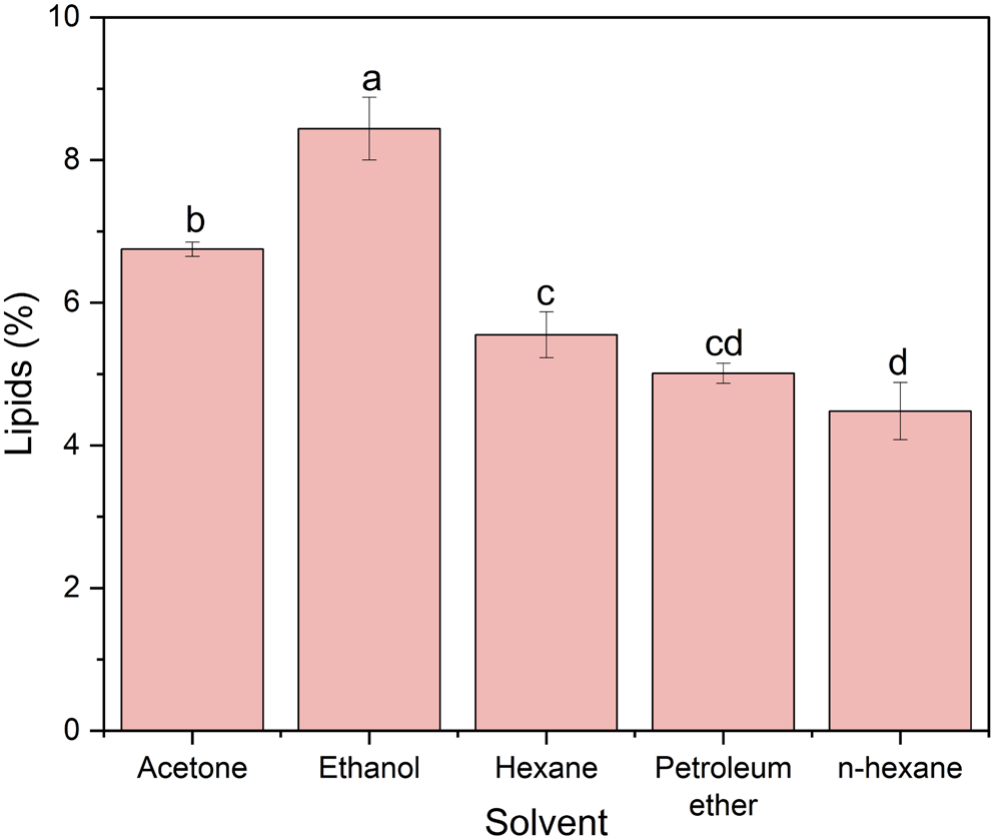
Effect of single solvent on lipids from brewers' spent grains (Sample size = 5 g; Solvent volume = 125 mL; Extraction time = 5 h).

The lipid contents extracted from BSG were significantly influenced by the choice of solvent. Figure 1 illustrates the lipid yields extracted from the different single solvents.

The lowest lipid content of 4.54% was observed with petroleum ether, while ethanol extracted the highest yield at 8.44%. Acetone, a moderately polar solvent, resulted in the extraction of 6.75% lipids. No significant differences were observed from hexane, n‐hexane, and petroleum ether in the lipid extraction yield, suggesting that non‐polar solvents may be less effective in extracting all lipids from BSG. Comparing these findings with previous studies on the extraction of lipids from BSG using the Soxhlet method is challenging due to variations in solvent selection and extraction conditions. However, Ortiz et al. (2015) reported a similar lipid yield of 8.6% lipids using 150 mL ethanol for an extraction time of 4 h at 80°C. In a recent study, Sanches et al. (2023) found lipid yields of 8.37% and 8.33% for “Lager” and “Weiss” styles of BSG, respectively, using ethanol and 24 h extraction in a Soxhlet extractor where the particle size was < 595 μm. A higher yield of 13.35% was obtained by Tadesse et al. (2025) when the authors extracted lipids from 0.3 mm BSG with ethanol as the solvent for 4 h. In the case of acetone, Herbst et al. (2021) reported 6.6% lipids using the Soxhlet method in BSG with 4.9% moisture content, while del Río et al. (2013) observed a higher yield of 9.2%. Hexane and n‐hexane are commonly used in Soxhlet extraction of BSG lipids. The 5.55% lipid yield from this study aligns with values reported by Ferrentino et al. (2019) for BSG with 7.1% moisture, Kavalopoulos et al. (2021) for BSG with 3.3% moisture, and Alonso‐Riaño et al. (2022) for BSG with 8.5% moisture, who found yields of 6.10%, 5.96%, and 5.92%, respectively. However, the results of the current study are slightly lower than those reported by Mallen and Najdanovic‐Visak (2018) and Vieira et al. (2014), who found yields of 7.50% at 68°C and in a range from 6.90% to 7.90%, respectively, for the extraction of lipids from BSG using n‐hexane. For petroleum ether, the yield of 5.01% obtained in the present study falls within values of 2.79% and 6.35% lipids extracted from BSG by Ajanaku et al. (2011) for 6.14% moisture and 610 μm particle size and Bonifácio‐Lopes et al. (2020), respectively. In a very recent study, a lipid yield of 4.82%–6.52% was measured for BSG from different beers when the authors used the Soxhlet method with petroleum ether for 5 h (García et al. 2025). In another study using petroleum ether, Nicolai et al. (2025) extracted 4.45% lipids from 400 μm BSG after 8 h extraction.

The selection of a single solvent for the extraction of all lipids in a food matrix is very complicated due to the heterogeneity of the samples. Previous studies have focused on characterizing the BSG in terms of the composition in which an accurate estimation of lipids might not be critically important. However, reference methods like Soxhlet are primarily used to develop calibration methods for instrument‐based methods. Thus, the solvent selection for lipid extraction must be carefully considered. The ideal solvent for lipid extraction should possess key properties such as high solvent power for lipids, ease of evaporation, low boiling point, non‐flammability, and non‐toxicity (Ellefson 2017). However, identifying a single solvent that meets all the above‐mentioned criteria is highly challenging. In contrast, some reference methods specify the use of a single solvent. For example, AOAC method 920.30 recommends anhydrous ether for crude fat determination in feed materials, while AOAC method 945.16 (AOAC 2016) suggests petroleum ether. Both solvents are non‐polar and highly flammable. Similarly, AACC method 30‐20.01(AACC International n.d.) for crude fat analysis in grains and feedstocks specifies dry ethyl ether for solvent. Thus, these two solvents may be less effective for sample matrices containing a broad spectrum of lipid types, including both polar and non‐polar lipids.

Several previous studies have investigated the effect of solvent choice on lipid extraction in the Soxhlet method. Ramluckan et al. (2014) evaluated 13 different solvents for extracting lipids from algal biomass and found that ethanol, chloroform, and hexane yielded the highest lipid recoveries. Although the primary objective of a study conducted by Herbst et al. (2021) was to compare pressurized liquid extraction with Soxhlet, they also reported that ethanol produced the highest lipid yield from BSG compared to ethyl acetate, acetone, and n‐hexane, a trend consistent with the findings from the present study. The superior performance of ethanol is likely due to the relatively high proportion of total polar lipids present in BSG. Supporting this, Lordan et al. (2019) reported that 38.9% of total lipids in BSG were polar. In their study, total lipids were extracted using the Bligh and Dyer method, and polar lipids and neutral lipids were separated via counter‐current distribution.

Ethanol is particularly effective for lipid extraction from BSG due to its high polarity index (PI) of 4.3, compared to petroleum ether and hexane, which have a PI of only 0.1. Ethanol polarity enables it to extract both polar and non‐polar lipids (Escorsim et al. 2018). Non‐polar solvents such as petroleum ether and hexane primarily solubilize long hydrophobic chains of fatty acids and neutral lipids through van der Walls interaction. In contrast, polar solvents like ethanol can solubilize polar lipids associated with cell walls via electrostatic interactions and hydrogen bonding (Halim et al. 2012). Moreau et al. (2003) also observed increased lipid yields with increasing solvent polarity in both corn and oats, with ethanol consistently producing the highest yield compared to hexane at 40°C and 100°C.

### 3.2 Effect of Binary Mixture

**FIGURE 2:**
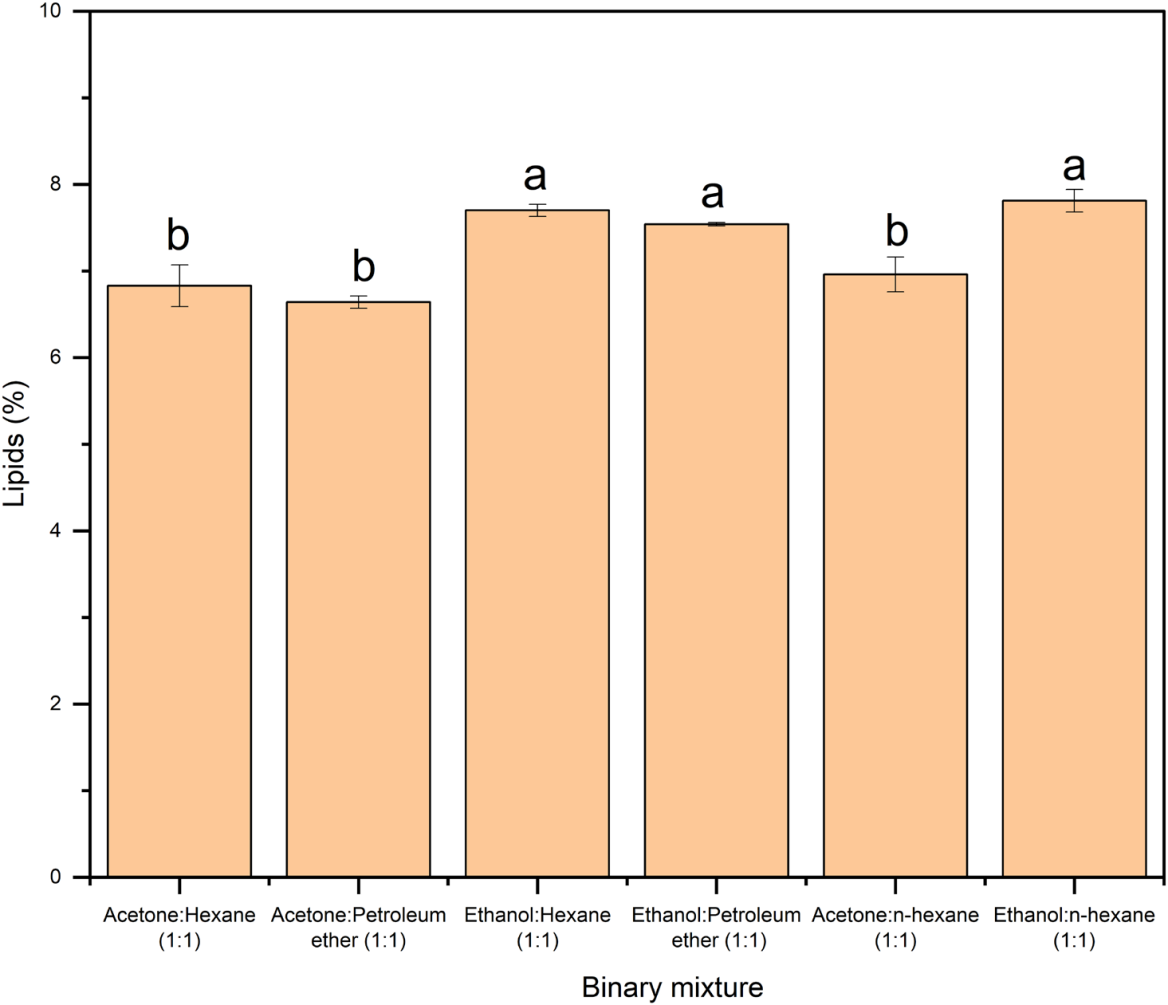
Effect of binary mixtures on lipids from brewers' spent grains (Sample size = 5 g; Binary mixture volume = 125 mL; Extraction time = 5 h).

Since a single solvent might not sufficiently extract all lipids from a sample matrix, the effect of solvent mixtures at a ratio of 1:1 was investigated. Based on their polarity indices, each polar solvent was combined with a non‐polar solvent, resulting in six different combinations. Figure 2 shows the lipid content extracted from BSG using these mixtures under conditions mentioned previously.

A statistical analysis revealed that combining ethanol with other non‐polar solvents resulted in the highest lipid yield compared to other solvent mixtures. Among the three ethanol‐based combinations, there was no significant difference in lipid content, which ranged from 7.64% to 7.81%. The values were slightly lower than the 8.44% lipid yield using ethanol alone in phase one of the study. This slight reduction may be attributed to the partial replacement of ethanol with a non‐polar solvent, which may not effectively remove the polar component of lipids in BSG. Results of the present study agree with a previous study by Ramluckan et al. (2014) who also noted that the extraction efficiencies of single solvents and 1:1 solvent mixtures were comparable for lipid extraction from algal biomass using the Soxhlet method.

An increase in the lipids yield with the addition of ethanol in binary mixtures may be due to viscosity effects. Enhanced solubility and improved transport of lipids through the cell wall are influenced by enthalpic and entropic interactions, which reduce the system's Gibbs free energy (Johnson and Lucas 1983). In addition to viscosity, the miscibility of organic solvents must also be considered when using solvent mixtures. Zarrinmehr et al. (2022) reported a decrease in lipid yield from microalgae when using two, three, and four solvent mixtures, attributing this to solvent miscibility. For instance, methanol was immiscible with chloroform and hexane, whereas acetone was miscible with both. They also observed higher lipid extraction efficiency with polar and non‐polar solvent mixtures compared to single polar solvents, findings that differ from those of the present study. A possible explanation for the similar yields between ethanol and ethanol‐based binary mixtures used in the present study is the distinct lipid composition of BSG compared to microalgae. For example, Escorsim et al. (2018) reported that using an ethanol‐hexane mixture for fat extraction from *Acutodesmus obliquus* increased the yield by 24% compared to ethanol alone, and by 217% compared to hexane alone. Similarly, Li et al. (2014) observed a three‐fold increase in the lipid yield for the marine microalga *Tetraselmis* when using a 3:1 hexane to ethanol ratio compared to using hexane alone. Thus, findings from these studies suggest that the effectiveness of a single solvent versus a binary mixture depends on the composition of the food matrix. In some cases, a single solvent may perform more efficiently than a binary mixture, and vice‐versa.

Based on the lipid yields observed in both phases 1 and 2, ethanol, ethanol:hexane, and ethanol:n‐hexane were selected for further investigation into the effect of extraction time.

### 3.3 Effect of Extraction Time

**FIGURE 3:**
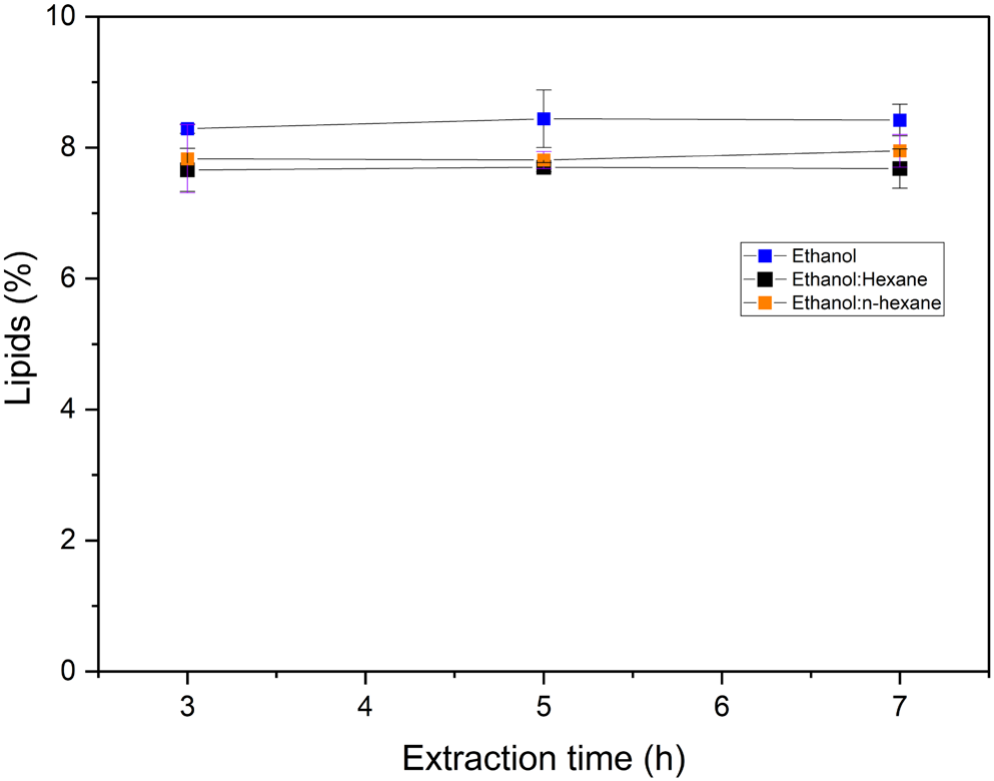
Effect of extraction time on lipids from brewers' spent grains (Sample size = 5 g; Solvent or binary mixture volume = 125 mL).

Figure 3 shows the lipid content extracted from BSG as a function of time for the selected solvent and solvent mixtures.

Under the experimental conditions tested (3–7 h), the effect of extraction time did not have a statistically significant effect on lipid yield (*p* > 0.05). The lipid yields ranged from 7.66% (ethanol: hexane at 3 h) to 8.44% (ethanol at 5 h). Ethanol consistently has higher lipid yields compared to the binary mixtures of ethanol:hexane and ethanol:n‐hexane. These results align with those of Ortiz et al. (2015) who reported a lipid yield of 8.6% from BSG using ethanol with a 4‐h extraction. The lipid yields from BSG in the current study are very similar to a study by Sanches et al. (2023), although these authors used ethanol extraction for 24 h compared to only 3 h here. In a recent study, Chu et al. (2025) extracted fatty acids from BSG using the Soxhlet method with a 20:50:1 ratio (n‐hexane/diethyl ether/formic acid) for 5 h, although the lipid yield was not reported.

Many previous studies support the findings of the current study in which the lipid yield reached a maximum value within the first few hours and a further increase in the extraction time did not necessarily increase the lipid yield. Ramluckan et al. (2014) observed that lipid yield from algal biomass increased until 3 h and then plateaued between 3 and 4 h when ethanol was used, closely following a similar trend to the one observed in the current study. Suwari et al. (2017) also reported a similar pattern for lipid extraction from seed kernel of Feun Kase using Soxhlet extraction with petroleum ether: methanol (90:10) where the lipid yield increased until 4.5 h and then leveled off until 6 h. In a study by Wang et al. (2005), the lipid yield was 7.26% within the first 30 min, 1.95% for the next 5.5 h (from 0.5 to 6 h), and only 0.11% for the next 6 h (from 6 to 12 h) when sorghum distiller dried grains (DDG) were subjected to lipid extraction using the Soxhlet method with n‐hexane as the solvent. A similar result was observed by Patel and Walker (2004), who did not observe an increase in lipid yield between 6 and 10 h for rice bran in the Soxhlet method when using petroleum ether as the solvent. A possible explanation is that the solvents used in the study were efficient enough to extract the maximum lipid content within the first few hours and extending that extraction time did not significantly improve the yield. The Soxhlet method is particularly effective in extracting the lipids due to the continuous reflux of hot solvent, which enhances both heat and mass transfer through the sample matrix. This finding emphasizes the importance of choosing the appropriate solvent in the Soxhlet method that can significantly reduce the extraction time in lipid extraction.

However, contrasting results have also been reported in the literature for lipid yield in terms of extraction time. Escorsim et al. (2018) observed a continuous increase in lipid yield from *Acutodesmus obliquus* when the extraction time was increased from 1 to 12 h using an ethanol:hexane (2:1) mixture. Li et al. (2014) found that lipid yield content from rapeseeds increased with extraction time from 0 to 6 h when using ethanol. In contrast, when using n‐hexane, the yield peaked at 3 h and declined thereafter. Ramluckan et al. (2014) also reported a decrease in lipid yield between 3 and 5 h for hexane and chloroform, attributing it to the formation of degradation products that reduced extractable lipids in algal biomass. In another study involving the Soxhlet method, the lipid yield reached the highest value at 8 h of extraction using n‐hexane, and then it slightly decreased between 8 and 25 h for spent coffee grounds (Efthymiopoulos et al. 2019). These findings suggest that the relationship between lipid yield and extraction time is highly dependent on both the sample matrix and the solvent system used in the process.

Accurate lipid determination is essential for food labeling and formulation. Many industry laboratories rely on rapid, instrument‐based methods using standard wet chemistry techniques. Our findings provide standardized Soxhlet parameters that ensure accurate lipid quantification in BSG, supporting calibration protocols and improving reliability in spectroscopic methods.

## 4 Conclusions

There is a growing need to standardize reference methods for measuring key components in foods, which is essential for developing accurate and robust prediction models using instrument‐based techniques such as vibrational spectroscopy. In this study, two key parameters of the Soxhlet method, solvent type, and extraction time, were standardized for the measurement of lipids in BSG, a byproduct with significant potential to be used as a dietary fiber‐rich ingredient in food formulations. Based on the results obtained, ethanol as a solvent and an extraction time of 3 h were found to yield the maximum lipid from BSG. Ethanol is regarded as a safer alternative to non‐polar solvents like hexane and petroleum ether and is also considered as a green solvent. Although the Soxhlet method typically involves the use of solvents, these can be effectively recycled through rotary evaporation. This study employed a one factor at a time approach to standardize the solvent type and extraction time. A key limitation of this method is that potential interactions between these factors were not considered. Additionally, factors such as BSG particle size and the sample to solvent volume ratio were not examined, even though they may affect lipid recovery during Soxhlet extraction. Future studies should investigate these factors using multifactorial experimental designs. To further characterize the extracted lipids, future studies should include analyzing the fatty acids profile using gas chromatography coupled with fatty acids methyl ester (FAME) analysis. Since BSG contains approximately 10% lipids, it represents a potential source of valuable compounds. Therefore, assessing lipid quality indicators such as primary and secondary oxidation values, sensory attributes (e.g., odor and color), and biological properties will provide a comprehensive understanding of its industry potential. This technique will allow for the precise identification and quantification of individual fatty acids, which is essential for evaluating health benefits, stability, and potential applications of BSG‐derived lipids to use in food formulations.

## Author Contributions

**Veeramani Karuppuchamy:** conceptualization, methodology, investigation, writing – original draft, writing – review and editing. **Osvaldo Campanella:** writing – review and editing, supervision, project administration, funding acquisition.

## Funding

This work was supported by the Carl E. Haas Endowment, GF602256; Sustainability Institute, Ohio State University, GF312903.

## Ethics Statement

The authors have nothing to report.

## Conflicts of Interest

The authors declare no conflicts of interest.

## Data Availability

The data that support the findings of this study are available on reasonable request from the corresponding author.

## References

[fsn371398-bib-0003] AACC International . n.d. Approved Methods of Analysis, 11th Ed. St. Paul, MN.

[fsn371398-bib-0001] Ajanaku, K. O. , F. A. Dawodu , C. O. Ajanaku , and O. C. Nwinyi . 2011. “Functional and Nutritional Properties of Spent Grain Enhanced Cookies.” American Journal of Food Technology 6, no. 9: 763–771. 10.3923/ajft.2011.763.771.

[fsn371398-bib-0002] Alonso‐Riaño, P. , R. Melgosa , E. Trigueros , A. E. Illera , S. Beltrán , and M. T. Sanz . 2022. “Valorization of Brewer's Spent Grain by Consecutive Supercritical Carbon Dioxide Extraction and Enzymatic Hydrolysis.” Food Chemistry 396: 133493. 10.1016/j.foodchem.2022.133493. 35879111

[fsn371398-bib-0004] Association of Official Analytical Chemists (AOAC) . 2016. AOAC International. Gaithersburg.

[fsn371398-bib-0005] Becker, D. , T. Bakuradze , M. Hensel , S. Beller , C. C. Yélamos , and E. Richling . 2021. “Influence of Brewer's Spent Grain Compounds on Glucose Metabolism Enzymes.” Nutrients 13, no. 8: 2696. 10.3390/nu13082696. 34444856 PMC8399999

[fsn371398-bib-0006] Bohnsack, C. , W. Ternes , A. Büsing , and A. M. Drotleff . 2011. “Tocotrienol Levels in Sieving Fraction Extracts of Brewer's Spent Grain.” European Food Research and Technology: Zeitschrift FüR Lebensmittel‐Untersuchung Und—Forschung A 232, no. 4: 563–573. 10.1007/s00217-010-1419-z.

[fsn371398-bib-0007] Bonifácio‐Lopes, T. , A. A. Vilas Boas , E. R. Coscueta , et al. 2020. “Bioactive Extracts From Brewer's Spent Grain.” Food & Function 11, no. 10: 8963–8977. 10.1039/d0fo01426e. 33001088

[fsn371398-bib-0008] Chu, H.‐Y. I. , T. Miri , and H. Onyeaka . 2025. “Valorization of Bioactive Compounds Extracted From Brewer's Spent Grain (BSG) for Sustainable Food Waste Recycling.” Sustainability 17, no. 6: 2477. 10.3390/su17062477.

[fsn371398-bib-0009] Connolly, A. , C. O. Piggott , and R. J. FitzGerald . 2013. “Characterisation of Protein‐Rich Isolates and Antioxidative Phenolic Extracts From Pale and Black Brewers' Spent Grain.” International Journal of Food Science & Technology 48, no. 8: 1670–1681. 10.1111/ijfs.12137.

[fsn371398-bib-0010] de Crane d'Heysselaer, S. , L. Bockstal , N. Jacquet , Q. Schmetz , and A. Richel . 2022. “Potential for the Valorisation of Brewer's Spent Grains: A Case Study for the Sequential Extraction of Saccharides and Lignin.” Waste Management & Research: The Journal of the International Solid Wastes and Public Cleansing Association, ISWA 40, no. 7: 1007–1014. 10.1177/0734242X211055547. 34713756

[fsn371398-bib-0011] del Río, J. C. , P. Prinsen , and A. Gutiérrez . 2013. “Chemical Composition of Lipids in Brewer's Spent Grain: A Promising Source of Valuable Phytochemicals.” Journal of Cereal Science 58, no. 2: 248–254. 10.1016/j.jcs.2013.07.001.

[fsn371398-bib-0012] Efthymiopoulos, I. , P. Hellier , N. Ladommatos , A. Kay , and B. Mills‐Lamptey . 2019. “Effect of Solvent Extraction Parameters on the Recovery of Oil From Spent Coffee Grounds for Biofuel Production.” Waste and Biomass Valorization 10, no. 2: 253–264. 10.1007/s12649-017-0061-4. 30873245 PMC6383743

[fsn371398-bib-0013] Ellefson, W. C. 2017. “Fat Analysis.” In Food Analysis, edited by S. S. Nielsen , 299–314. Springer International Publishing. 10.1007/978-3-319-45776-5_17.

[fsn371398-bib-0014] Escorsim, A. M. , G. da Rocha , J. V. C. Vargas , et al. 2018. “Extraction of *Acutodesmus obliquus* Lipids Using a Mixture of Ethanol and Hexane as Solvent.” Biomass & Bioenergy 108: 470–478. 10.1016/j.biombioe.2017.10.035.

[fsn371398-bib-0015] Ferrentino, G. , J. Ndayishimiye , N. Haman , and M. Scampicchio . 2019. “Functional Activity of Oils From Brewer's Spent Grain Extracted by Supercritical Carbon Dioxide.” Food and Bioprocess Technology: An International Journal 12, no. 5: 789–798. 10.1007/s11947-019-02249-3.

[fsn371398-bib-0016] García, D. C. , I. Villalba , N. Savino , and M. A. Nazareno . 2025. “Nutritional and Functional Characterization of Different Types of Brewer's Spent Grain Flours.” Food Bioscience 64: 105890. 10.1016/j.fbio.2025.105890.

[fsn371398-bib-0017] Guarda, E. C. , A. C. Oliveira , S. Antunes , et al. 2021. “A Two‐Stage Process for Conversion of Brewer's Spent Grain Into Volatile Fatty Acids Through Acidogenic Fermentation.” Applied Sciences 11: 3222. 10.3390/app11073222.

[fsn371398-bib-0018] Halim, R. , R. Harun , M. K. Danquah , and P. A. Webley . 2012. “Microalgal Cell Disruption for Biofuel Development.” Applied Energy 91, no. 1: 116–121. 10.1016/j.apenergy.2011.08.048.

[fsn371398-bib-0019] Herbst, G. , F. Hamerski , M. Errico , and L. Corazza 2021. “Pressurized Liquid Extraction of Brewer's Spent Grain: Kinetics and Crude Extracts Characterization.” Journal of Industrial and Engineering Chemistry 102: 370–383. 10.1016/j.jiec.2021.07.020.

[fsn371398-bib-0020] Johnson, L. A. , and E. W. Lucas . 1983. “Comparison of Alternative Solvents for Oils Extraction.” Journal of the American Oil Chemists' Society 60: 229–242. 10.1007/BF02543490.

[fsn371398-bib-0021] Karuppuchamy, V. , D. R. Heldman , and A. B. Snyder . 2024. “A Review of Food Safety in Low‐Moisture Foods With Current and Potential Dry‐Cleaning Methods.” Journal of Food Science 89, no. 2: 793–810. 10.1111/1750-3841.16920. 38221802

[fsn371398-bib-0022] Kavalopoulos, M. , V. Stoumpou , A. Christofi , et al. 2021. “Sustainable Valorisation Pathways Mitigating Environmental Pollution From Brewers' Spent Grains.” Environmental Pollution 270: 116069. 10.1016/j.envpol.2020.116069. 33338956

[fsn371398-bib-0023] Li, Y. , F. Ghasemi Naghdi , S. Garg , et al. 2014. “A Comparative Study: The Impact of Different Lipid Extraction Methods on Current Microalgal Lipid Research.” Microbial Cell Factories 13, no. 1: 13–14. 10.1186/1475-2859-13-14. 24456581 PMC3926349

[fsn371398-bib-0024] Lordan, R. , E. O'Keeffe , A. Tsoupras , and I. Zabetakis . 2019. “Total, Neutral, and Polar Lipids of Brewing Ingredients, By‐Products and Beer: Evaluation of Antithrombotic Activities.” Food 8, no. 5: 171. 10.3390/foods8050171. PMC6560433 31137500

[fsn371398-bib-0025] Madubuike, P. C. , and T. C. Okolo . 2016. “Quality Estimation of Brewer's Spent Grains and Its Potential: A Product of Beer Industries.” International Journal of Engineering Science 5, no. 3: 21–25.

[fsn371398-bib-0026] Mallen, E. , and V. Najdanovic‐Visak . 2018. “Brewers' Spent Grains: Drying Kinetics and Biodiesel Production.” Bioresource Technology Reports 1: 16–23. 10.1016/j.biteb.2018.01.005.

[fsn371398-bib-0027] Moreau, R. A. , M. J. Powell , and V. Singh . 2003. “Pressurized Liquid Extraction of Polar and Nonpolar Lipids in Corn and Oats With Hexane, Methylene Chloride, Isopropanol, and Ethanol.” Journal of the American Oil Chemists' Society 80, no. 11: 1063–1067. 10.1007/s11746-003-0821-y.

[fsn371398-bib-0028] Moreira, M. M. , S. Morais , A. A. Barros , C. Delerue‐Matos , and L. F. Guido . 2012. “A Novel Application of Microwave‐Assisted Extraction of Polyphenols From Brewer's Spent Grain With HPLC‐DAD‐MS Analysis.” Analytical and Bioanalytical Chemistry 403, no. 4: 1019–1029. 10.1007/s00216-011-5703-y. 22274285

[fsn371398-bib-0029] Mussatto, S. I. , G. Dragone , and I. C. Roberto . 2006. “Brewers' Spent Grain: Generation, Characteristics and Potential Applications.” Journal of Cereal Science 43: 1–14. 10.1016/j.jcs.2005.06.001.

[fsn371398-bib-0030] Nicolai, M. , M. L. Palma , R. Reis , et al. 2025. “Assessing the Potential of Brewer's Spent Grain to Enhance Cookie Physicochemical and Nutritional Profiles.” Food 14, no. 1: 95. 10.3390/foods14010095. PMC11719959 39796385

[fsn371398-bib-0031] Niemi, P. , T. Tamminen , A. Smeds , et al. 2012. “Characterization of Lipids and Lignans in Brewer's Spent Grain and Its Enzymatically Extracted Fraction.” Journal of Agricultural and Food Chemistry 60, no. 39: 9910–9917. 10.1021/jf302684x. 22963516

[fsn371398-bib-0032] Ortiz, S. , A. A. C. Barros , and A. Furigo Jr. 2015. “Extraction and Quantification of Lipids From Brewer's Spent Grain and Its Potential for Lipase Production.” Blucher Chemical Engineering Proceedings 1, no. 2: 1–7. 10.5151/chemeng-cobeq2014-2071-15995-146531.

[fsn371398-bib-0033] Parekh, I. , A. Khanvilkar , and A. Naik . 2017. “Barley‐Wheat Brewers' Spent Grain: A Potential Source of Antioxidant Rich Lipids.” Journal of Food Processing and Preservation 41, no. 6: 13244. 10.1111/jfpp.13244.

[fsn371398-bib-0034] Patel, P. , and T. Walker . 2004. “Effect of Extraction Method on the Level of Important Antioxidants in Rice Bran Oil.” ASAE Annual Meeting: 46140. 10.13031/2013.17701.

[fsn371398-bib-0035] Pires, E. J. , H. A. Ruiz , J. A. Teixeira , and A. A. Vicente . 2012. “A New Approach on Brewer's Spent Grains Treatment and Potential Use as Lignocellulosic Yeast Cells Carriers.” Journal of Agricultural and Food Chemistry 60, no. 23: 5994–5999. 10.1021/jf300299m. 22624780

[fsn371398-bib-0036] Ramluckan, K. , K. G. Moodley , and F. Bux . 2014. “An Evaluation of the Efficacy of Using Selected Solvents for the Extraction of Lipids From Algal Biomass by the Soxhlet Extraction Method.” Fuel 116: 103–108. 10.1016/j.fuel.2013.07.118.

[fsn371398-bib-0037] Sanches, M. A. R. , P. E. D. Augusto , T. C. Polachini , and J. Telis‐Romero . 2023. “Water Sorption Properties of Brewer's Spent Grain: A Study Aimed at Its Stabilization for Further Conversion Into Value‐Added Products.” Biomass & Bioenergy 170: 106718. 10.1016/j.biombioe.2023.106718.

[fsn371398-bib-0038] Santos, M. , J. J. Jiménez , B. Bartolomé , C. Gómez‐Cordovés , and M. J. del Nozal . 2003. “Variability of Brewer's Spent Grain Within a Brewery.” Food Chemistry 80, no. 1: 17–21. 10.1016/S0308-8146(02)00229-7.

[fsn371398-bib-0039] Suwari , H. Z. Kotta , Y. Buang , et al. 2017. “Optimization of Soxhlet Extraction and Physicochemical Analysis of Crop Oil From Seed Kernel of Feun Kase ( *Thevetia peruviana* ).” AIP Conference Proceedings 1911, no. 1: 998. 10.1063/1.5015998.

[fsn371398-bib-0040] Tadesse, H. M. , T. Atnafu , E. Kassahun , I. Tessema , M. Abewaa , and S. Tibebu . 2025. “Optimization of Bioethanol Production From a Brewers' Spent Grain and Sugarcane Molasses Mixture Utilizing *Saccharomyces cerevisiae* .” Biomass Conversion and Biorefinery 15, no. 14: 20765–20788. 10.1007/s13399-025-06629-y.

[fsn371398-bib-0041] Vieira, E. , M. A. M. Rocha , E. Coelho , et al. 2014. “Valuation of Brewer's Spent Grain Using a Fully Recyclable Integrated Process for Extraction of Proteins and Arabinoxylans.” Industrial Crops and Products 52: 136–143. 10.1016/j.indcrop.2013.10.012.

[fsn371398-bib-0042] Vieira, E. F. , D. D. da Silva , H. Carmo , and I. M. P. L. V. O. Ferreira . 2017. “Protective Ability Against Oxidative Stress of Brewers' Spent Grain Protein Hydrolysates.” Food Chemistry 228: 602–609. 10.1016/j.foodchem.2017.02.050. 28317769

[fsn371398-bib-0043] Wang, L. , C. L. Weller , and K. T. Hwang . 2005. “Extraction of Lipids From Grain Sorghum DDG.” Transactions of ASAE 48, no. 5: 1883–1888. 10.13031/2013.19986.

[fsn371398-bib-0044] Zarrinmehr, M. J. , E. Daneshvar , S. Nigam , et al. 2022. “The Effect of Solvents Polarity and Extraction Conditions on the Microalgal Lipids Yield, Fatty Acids Profile, and Biodiesel Properties.” Bioresource Technology 344: 126303. 10.1016/j.biortech.2021.126303. 34752885

